# Case Report: COVID-19 and Lassa Fever Coinfection in an Ebola Suspected Patient in Guinea

**DOI:** 10.4269/ajtmh.21-0713

**Published:** 2022-02-25

**Authors:** Mory Keita, Mahamoud Sama Cherif, Billy Sivahera, Samuel T. Boland, Freddy Banza-Mutoka, Mamadou Kourouma, Alseny Modet Camara, Youssouf Sidibe, Jean Paul Kimenyi, Lamine Diassy, Angelo Loua, Ibrahima Sory Fofana, Youba Kandako, Dobo Onivogui, Enogo Koivogui, Tamba Jacques Millimono, Fode Diakite, Mamadou Balde, Bienvenu Houndjo, Ngoy Nsenga, Ambrose Talisuna, Alexandre Delamou, Olivia Keiser, Georges Alfred Ki-Zerbo, Abdou Salam Gueye

**Affiliations:** ^1^ World Health Organization, Regional Office for Africa, Brazzaville, Congo;; ^2^ Institute of Global Health, Faculty of Medicine, University of Geneva, Geneva, Switzerland;; ^3^ Faculty of Sciences and Health Technics, Gamal Abdel Nasser University of Conakry, Conakry, Guinea;; ^4^ World Health Organization, Country Office for Guinea, Conakry, Guinea;; ^5^ Department of Global Health & Development, London School of Hygiene & Tropical Medicine, London, United Kingdom;; ^6^ Alliance for International Medical Action (ALIMA), Dakar, Senegal;; ^7^ Ministry of Health, Regional Hospital of Nzerekore, Department of Laboratory, Molecular Biology Unit;; ^8^ Ministry of Health, Prefectoral Health Directorate of Nzerekore, Guinea;; ^9^ Ministry of Health, Prefectoral Health Directorate of Yomou, Guinea;; ^10^ National Agency for Health Security of Guinea;; ^11^ Africa Center of Excellence (CEA-PCMT), University Gamal Abdel Nasser, Conakry, Guinea

## Abstract

In this case report, we describe a clinical presentation and therapeutic history of a unique case diagnosed with Lassa fever and severe acute respiratory syndrome coronavirus 2 (SARS-CoV-2) in a 23-year-old man from Yomou prefecture in southeast Guinea identified with suspected Ebola Virus Disease (EVD) in the midst of an ongoing outbreak of that disease in the same region. On May 3, 2021, he was admitted to the Nzérékoré Epidemic disease treatment center where his clinical condition deteriorated significantly. Laboratory testing performed on the same day reveals a negative EVD polymerase chain reaction (PCR). Three days later, the patient was tested positive for SARS-CoV-2 and Lassa fever by reverse transcriptase PCR (RT-PCR) assays. Laboratory examination also indicated severe hematological and biochemical deteriorations in the patient. This case substantiates the need for systematic differential diagnosis during epidemic-prone disease outbreaks to better manage severely unwell patients.

## INTRODUCTION

Emerging infectious diseases such as Ebola Virus Disease (EVD), COVID-19, and Lassa fever pose significant epidemic threats. ^1^^,^ ^2^

In Guinea, the ongoing COVID-19 pandemic has already resulted in more than 23,501 cases and 168 recorded deaths ^3^ (from March 12, 2020 to June 19, 2021). The country has also been facing the resurgence of EVD officially declared on February 14, 2021. ^4^ As of June 10, 2021, a total of 23 cases, including 16 confirmed cases (10 recovered and 5 deaths) and 7 probable cases, have been recorded in the district of Nzérékoré, located in the country’s southeast. ^5^

In addition to the ongoing COVID-19 pandemic and EVD outbreak and to further complicate matters, on May 17, 2021, the Ministry of Health (MoH) declared a Lassa fever epidemic in the Prefecture of Yomou.

Lassa fever is caused by the Lassa virus. It is a viral hemorrhagic fever (VHF) that is endemic in West Africa (primarily in Sierra Leone, Guinea, Liberia, and Nigeria ^6^). The primary reservoir host is the multimammate rodent *Mastomys nataliensis*, which often lives close to human populations. ^7^ In recent years, several advancements in Lassa fever identification have been made, such as improved diagnostics through real-time polymerase chain reaction (PCR) for genomic signatures and the successful utilization of sequencing technology to diagnose and characterize the virus. ^8^

The concurrent COVID-19, Ebola, and Lassa fever outbreaks pose unique and significant risks to local populations in Guinea, especially as the country’s situation is characterized by community reluctance (i.e., rejection of directions from national or international experts) and frustration following the drawn-out COVID-19 pandemic and the recent 2013–2016 West Africa Ebola Epidemic. Thoroughly understanding comorbidity is also paramount, there are no reports for human infection with Lassa fever and COVID-19 as of this report.

## CASE REPORT

A 23-year-old man from Yomou district in southeastern Guinea was identified with suspected EVD on May 3, 2021 at the Nzérékoré Regional Hospital. The patient presented with fever (38.8°C), anorexia, asthenia, chest pain, muscle pain, joint pain, abdominal pain, and insomnia. He was isolated, transferred, and admitted to the Ebola Treatment Center (ETC) the same day. Physical exam findings on admission included a temperature of 39.4°C, blood pressure of 170/100 mm of Hg, respiratory rate of 26 breaths/minute, blood oxygen saturation of 97.2%, and pulse rate of 100 beats/minute. Samples (blood and swabs) were collected and tested for acute viral infection. Polymerase chain reaction testing was performed using the Fosun COVID-19 reverse transcriptase PCR (RT-PCR) Detection Kit (Fosun Pharma, Shanghai City, China) to confirm the suspected COVID-19 infection. The Lassa fever was confirmed by RealStar^®^ Lassa Virus RT-PCR Kit (Altona Diagnostics, Hamburg, Germany). The Xpert^®^ Ebola (Cepheid, Sunnyvale, CA) was used to confirm the suspected EVD infection. The Marburg virus disease test was carried out using the RealStar filovirus RT-PCR Kit (Altona Diagnostics). While EVD and Marburg tests were negative, the patient was found to be Lassa fever positive and COVID-19 positive by PCR. Further laboratory testing (blood biochemical tests) showed that hepatic (increased levels of alanine aminotransferase [ALT] and aspartate aminotransferase [ASAT]) and kidney functions (an elevated creatinine level) were seriously altered and required immediate treatment (Table 1).

**Table 1:** Dynamic of hematology and biochemistry profile of the patient between admission 1 and 2

Laboratory	Reference range	First day of admission (May 3, 2021)	Second day of admission (May 7, 2021)
Hematology			
Hematocrit (%)	26.0–50.0	46.1	47.3
Hemoglobin (g/dL)	8.0–17.0	16.3	18.2
White blood cells (×10^3^/µL)	3.0–15.0	5.1	14.7
Mean corpuscular volume (fL)	86.0–110.0	79.9	74.5
Mean corpuscular hemoglobin (pg)	26.0–38.0	28.2	28.7
Neutrophils (%)	45.0–95.0	85.2	77.7
Lymphocytes (%)	5.0–55.0	12.1	15.1
Platelets (×10^3^/µL)	50–400	52	72
Biochemistry			
Glucose (mg/dL)	73–118	153	118
Blood urea nitrogen (mg/dL)	7–22	28	168
Creatinine (mg/dL)	0.6–1.2	2.1	15.3
T bilirubin (mg/dL)	0.2–1.6	0.9	4.4
Albumin (g/dL)	3.3–5.5	2.3	1.5
Aspartate transaminase (U/L)	11–38	1,018	> 2,000
Alanine transaminase (U/L)	10–47	288	1,344
Creatine kinase (U/L)	30–380	1,771	4,775
Sodium (mmol/L)	128–145	123	125
Potassium (mmol/L)	3.6–5.1	2.9	Very high (results falling outside of the highest display range set in the analyzer).
C-reactive protein (mg/L)	0.0–7.5	40.8	81.9

**Figure 1.**
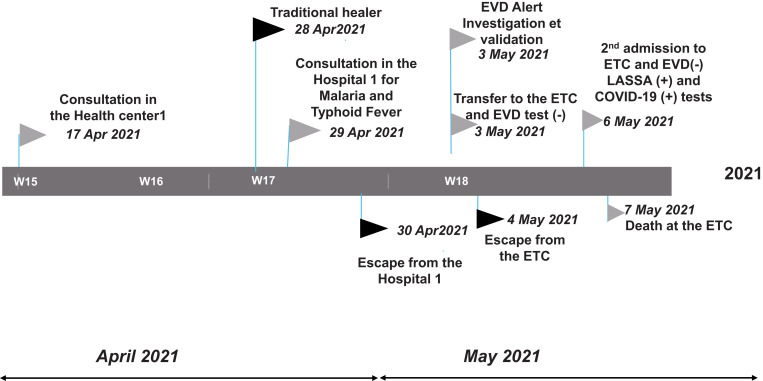
Therapeutic itinerary of the COVID-19 and Lassa fever coinfection patient from his village (the health district of Yomou) to the regional capital (Nzérékoré), April to May 2021.

On May 4, 2021, the patient left the Nzérékoré ETC of his own accord and without informing hospital staff. He returned to his village in Yomou. Upon being informed of the situation, a family member brought him to a local EVD Treatment Unit (ETU) where biosafety measures were put in place, and the patient was admitted. The patient was then transferred back to the Nzérékoré ETC for further testing and investigation (Figure 1).

On May 6, 2021, readmittance to the ETC was deemed necessary because of the appearance of new symptoms such as respiratory distress and the further deterioration of the patient’s clinical condition (asthenia, abdominal pain, and oliguria). More laboratory testing was conducted to guide medical actions.

Hematology performed with Sysmex pocH-100i^™^ Automated Hematology Analyzer (Sysmex Corporation, Kobe, Japan) and biochemistry with Piccolo Xpress^®^ (Abaxis, Inc., Union City, CA) showed a severe deterioration of hepatic, renal, and hematological functions (Table 1). The treatment plan consisted of rehydration, the provision of oxygen therapy and antibiotics, and other symptomatic treatments.

Despite attempts at several treatments including oxygen therapy and ultimately resuscitation, the patient rapidly deteriorated and died on May 7, 2021 with respiratory distress and organ failure symptoms.

To better understand the chains of transmission and to appreciate the potential risks and extent of the viruses’ circulation, the MoH collaborated with the WHO and other partners to carry out a thorough case investigation. This case investigation found 30 contacts and public health actions were initiated immediately. Further to few days’ rejection of response activities implementation, the community accepted the strategy of community confinement (i.e., no movement of contacts outside their village until the end of the follow-up period) with food support. Contact follow-up continued until the end of the 21-day period without any secondary cases being detected.

## DISCUSSION

Many cases of Lassa fever have been described with specific conditions. ^9^^–^ ^11^ However, the unusual association of two epidemic-prone diseases (Lassa fever and COVID-19) in the context of an ongoing Ebola outbreak is a unique and previously unreported case.

The case reported here was admitted to the ETC with symptoms that included those in the EVD case definition. ^11^ Additionally, he presented a respiratory distress syndrome that is common in Lassa fever (up to 20% of cases) ^12^^,^ ^13^ as well as in COVID-19. ^14^ Laboratory testing showed liver and kidney failure, which directed the clinical management team to conduct further testing as early identification of Lassa fever is one of the critical points for maximizing the benefit of further available care such as antiviral therapy. ^15^

The hematological analysis showed polycythemia in which the hematocrit and/or Hb concentration are elevated in peripheral blood and may reduce the blood flow and the amount of oxygen that reaches heart, brain, and other vital organs increasing the risk of clots within a blood vessel causing a stroke or death. ^16^^–^ ^18^ He had a high risk of vascular thrombosis, which is well described in patients dying of COVID-19. This supports the reported statement that the thrombotic complications are central determinants of the high mortality rate in COVID-19 and that thrombosis prevention strategies remain important. ^16^ The biochemical analyses carried out on May 3 and May 7, 2021 showed elevated levels of ALT and ASAT markers more than 100 time arguing for acute hepatitis. The kidney function marker measured by creatinine and blood urea nitrogen were at least increased seven times the upper limit of normal.

This case also clarifies the need for laboratory testing that can run differential diagnoses between various diseases that share the same symptoms and expression, especially those that are endemic, epidemic-prone, or contemporaneously pandemic. Since the case lived in a highly rural area of the district of Yomou where the reported frequency of COVID-19 is among the lowest in the country suggests that the COVID-19 incidence might be underestimated in African countries as several reports have suggested. ^19^^,^ ^20^

The reemergence of Lassa fever in Guinea was of grave concern. This patient twice left without notice from medical centers foregoing the opportunity for proper and timelier care. His subsequent return in a seriously deteriorated condition was too late for available care to prove effective. The patient’s source of infection remains unknown both for COVID-19 and Lassa fever. Moreover, subsequent confirmation of both diagnoses led to an in-depth investigation that revealed no evidence of COVID-19 coexisting cases with the patient during his first hospitalization and excluded any possible contamination that occurred there. Although Lassa fever is endemic in this area, ^6^^,^ ^21^ the source could be related to human-to-human or zoonotic transmission. The COVID-19 contamination may have occurred anywhere else given the spread of the disease in Guinea.

This event is a fundamental reminder that endemic and epidemic-prone diseases continue to occur during the COVID-19 pandemic affecting the capacities of exhausted healthcare professionals. ^22^^–^ ^24^ The first-ever outbreak of Marburg Virus Disease in the West African region was recently reported in Guinea, ^25^ few weeks after declaring the end of the EVD outbreak. ^26^

Cross-border issues may be a significant factor in this case as of May 25, 2021, a Lassa fever outbreak was ongoing in Liberia, and suggest an epidemiological link between the outbreaks that must be investigated and taken into account. ^20^^,^ ^21^ This case makes it clear how important it is to identify, track, and predict the spread of disease so that all levels of government can respond quickly and deploy the resources necessary to stop its spread.

Finally, as one epidemic can hide another, this case clarifies the need to expand disease-specific surveillance to areas surrounding the outbreak’s known geography. In this case, the extension of Ebola surveillance into neighboring districts allowed us to identify and diagnose this Lassa fever index case, facilitating the prompt declaration of a Lassa fever epidemic and the rapid implementation of control measures to limit the virus’ spread in Guinea and Liberia.
